# Analgesia Evaluation of 2 NSAID Drugs as Adjuvant in Management of Chronic Temporomandibular Disorders

**DOI:** 10.1155/2015/359152

**Published:** 2015-03-22

**Authors:** Fernando Kurita Varoli, Murillo Sucena Pita, Sandra Sato, João Paulo Mardegan Issa, Cássio do Nascimento, Vinícius Pedrazzi

**Affiliations:** ^1^Department of Dental Materials and Prosthodontics, Faculty of Dentistry of Ribeirão Preto, University of São Paulo, Avenida do Café s/no, Monte Alegre, 14040-904 Ribeirão Preto, SP, Brazil; ^2^Department of Morphology, Physiology, and Basic Pathology, Avenida do Café s/no, Monte Alegre, 14040-904 Ribeirão Preto, SP, Brazil

## Abstract

The aim of this triple-blind full-randomized clinical trial was to quantify analgesia in masticatory muscles and temporomandibular joints after occlusal splint therapy associated with the adjuvant administration of nonsteroidal anti-inflammatory drugs (NSAID) isolated or associated with other therapeutic agents. Pain relief was also recorded. Eighteen volunteers who had been suffering from chronic pain in masticatory muscles due to temporomandibular disorders were selected after anamnesis and assessment using RDC/TMD translated to Portuguese. The 3 proposed treatments were NSAID (sodium diclofenac), panacea (sodium diclofenac + carisoprodol + acetaminophen + caffeine), and a placebo. The total treatment duration was 10 days, preceded and succeeded by patients' pain assessment. A washout interval of 11 days was established between each therapy. All participants received all treatments in different moments, in a full randomized crossover methodology. The assessment of drug therapies was performed using visual analogue scale for pain on palpation followed by 11-point numerical scale to quantify pain during treatment. Statistical analysis has shown that, after 10 days of treatment, all therapies were effective for pain relief. NSAID therapy promoted analgesia on the third day, while placebo only promoted analgesia in the eighth day. It has been concluded that sodium diclofenac used as splint adjuvant therapy, promotes significant analgesia in a shorter time.

## 1. Introduction

Temporomandibular disorders (TMD) result from musculoskeletal dysfunction of the orofacial region affecting masticatory muscles, temporomandibular joints (TMJ), and other associated structures. The main characteristics of these problems are facial and TMJ pain, headache, earache, dizziness, masticatory muscle hypertrophy, limited mouth opening, locked jaw, abnormal teeth wear, joint sounds, and others [1]. Besides the wide variety of signs and symptoms, pain is the main reason leading the patient to search for treatment [2].

Dentist must be aware on the proper diagnosis and treatment of temporomandibular disorders, because they represent the second most frequent patients complaints (only less frequent than dental pain) [3]. The origin of these disorders is probably multifactor, resulting from a dynamic adaptive and destructive process [4]. Temporomandibular disorders pain may result from an inflammatory cause, mainly due to microlesions on the masticatory muscles and TMJ. About 44% to 79% of individuals report traumatic events affecting the orofacial region, including macro- and microtraumas from masticatory muscles overloaded, or repetitive loading and muscular fatigue [4]. These events can cause microlesions at masticatory muscle fibers leading to a local inflammatory mediators' release, such as prostaglandins, bradykinin, histamine, and substance P, which may induce or facilitate nociceptive afferent impulse transmission to superior nervous center, developing peripheral and central sensitization [5–7].

In the management of temporomandibular disorders, it is primordial to relief the pain, preventing relevant alterations in neuronal circuits and secondary hyperalgesia caused by persistent afferent signs [8] which is substantially modulated by psychological, behavioral, and psychosocial factors [9]. When peripheral events are not controlled, acute pain may become chronic, mainly due to patient vulnerability including genetic predisposition, hormonal factors, behavioral habits, and previous unsuccessful therapies. General disorder can present negative symptoms as depression, social isolation, indifference, inactivity, excessive health care use, and affliction, with reduced biomedical treatment response [4].

Structural and neurochemical alterations on the central nervous system associated with the presence of psychosocial factors increase and perpetuate painful sensation, making chronic pain a great challenge for clinicians [10]. In such case, nonsteroidal anti-inflammatory drugs could act inhibiting inflammatory mediators released in muscle tissues, reducing signs and symptoms, including pain.

Thus, the aim of this full randomized triple-blind crossover study was to quantify analgesia obtained by NSAID, associated or not with other therapeutic agents, in patients diagnosed with chronic muscle pain. The null hypothesis is that NSAID may reduce or eliminate pain resulted that from TMD.

## 2. Material and Methods

### 2.1. Participant's Selection

All the clinical and laboratorial steps were performed by only one researcher, to avoid interexaminer differences, standardizing procedures, and reducing result bias.

Eighteen adult volunteers, both men and women, between 35 and 70 years of age (mean age 50 years), who were included in a maintenance-care program at the Faculty of Dentistry of Ribeirão Preto, University of São Paulo, were enrolled in this study. The inclusion criteria were masticatory muscle pain. The exclusion criteria included pregnancy, lactation, anti-inflammatory, or antibiotic therapy within the previous 3 months, current smokers, or individuals with any systemic condition that could influence pain management. The local ethics committee approved this study, and all of the experiments were undertaken with the informed written consent of each participant in accordance with ethical principles (Lawsuit n. 2006.1.558.58.0, CAAE n. 0022.0.138.000-06).

A complete patient trial was carried out by using an anamnesis with questions regarding general health. There were also excluded participants on this study with (1) under 18 years old; (2) who worn removable dental prosthesis; (3) who were taking any other medicament; (4) whose health did not allow intake of those studied drugs; and (5) alcohol addict.

The Brazilian Portuguese version of Research Diagnostic Criteria of Temporomandibular Disorders (RDC/TMD) [11] was used in trial.

### 2.2. Experimental Design (Treatment Protocol)

All participant enrolled in this investigation received a flat, full-covered, and rigid occlusal splint adjusted in centric relation contact position, with canine guides and simultaneous occlusal contacts. Patients were advised to use the occlusal splint during all the 10 days of investigation. The provoked pain was performed by finger pressure on both sides of masseter, temporalis, sternocleidomastoideus, trapezius, and TMJ lateral pole. Data from intensity of pain was assessed on the first and last day of study. Patients were instructed to mark on 10 mm visual analogue scales (VAS) the pain intensity of each examined site. In addition, during treatment period, patients were instructed to mark at an 11-point numeric scale, from 0 (absence of pain) up to 10 (the worst pain ever), once a day during nocturnal period, the value related pain intensity of that day.

During treatment, patients were instructed to use the 3 treatment protocols proposed in this investigation: (a) NSAID (sodium diclofenac), (b) panacea (sodium diclofenac + carisoprodol + acetaminophen + caffeine), and (c) Placebo. A full randomized crossover methodology was adopted and, in this manner, all patients were submitted to all treatments in different moments. Therefore, the sequence of treatments did not follow a standardized sequence, avoiding bias. A washout interval of 11 days was respected between each proposed protocol.

The drugs used in this study are displayed in Table 1, including composition, active ingredients, concentration, manufacturer, and lot number. Medicaments A and B were acquired in pharmacy stores. Medicament C was manufactured in the Faculty of Pharmaceutical Sciences of Ribeirão Preto (University of São Paulo, Brazil) using a capsule shape.

During treatment, individuals were informed to take two daily medicament dosages, according to the manufacturer's recommendation, during 10 days, followed by an 11-day washout interval, started after the last dosage of each drug. All patients were instructed not to wear occlusal splint and not take any medicament during washout period.

### 2.3. Data Analysis

Statistical analysis was performed by an investigator not informed about medicaments compositions. Normality tests (Kolmogorov-Smirnov) were performed for the provoked pain data obtained by VAS. Most data did not present Gaussian distribution, and then a logarithm transformation was done. A 0.5 constant was added to all pain scores, to allow logarithm be used on zero scores [12]. After that, data normalization was obtained, allowing repeated measures by ANOVA.

For daily pain scores, statistical analysis was performed using nonparametric statistic methods. Friedman's test was applied to investigate pain value differences among each day of treatment. When differences were found, Wilcoxon's test was performed to compare the initial pain score to each subsequent treatment day. For both tests, significant differences were considered when *P* < 0.05.

## 3. Results

**Figure 1:**
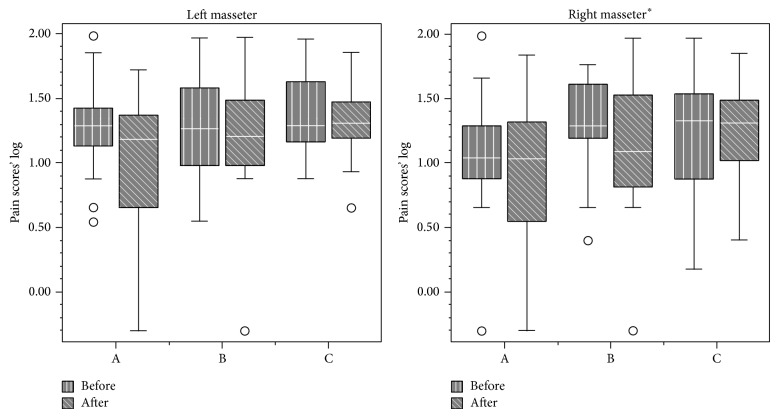
Pain scores (median and interquartile intervals) before and after treatment in the masseter muscle from groups A (NSAID—sodium diclofenac), B (panacea—sodium diclofenac + carisoprodol + acetaminophen + caffeine), and C (placebo). (^*^Significant differences; *P* < 0.05).

**Figure 2:**
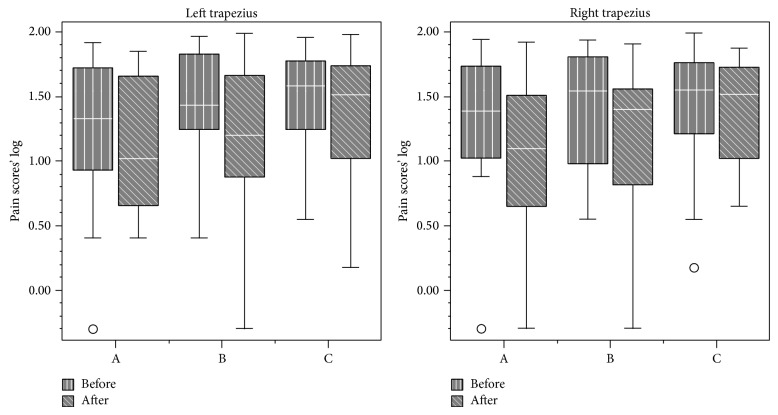
Pain scores (median and interquartile intervals) before and after treatment in the trapezius muscle from groups A (NSAID—sodium diclofenac), B (panacea—sodium diclofenac + carisoprodol + acetaminophen + caffeine), and C (placebo).

**Figure 3:**
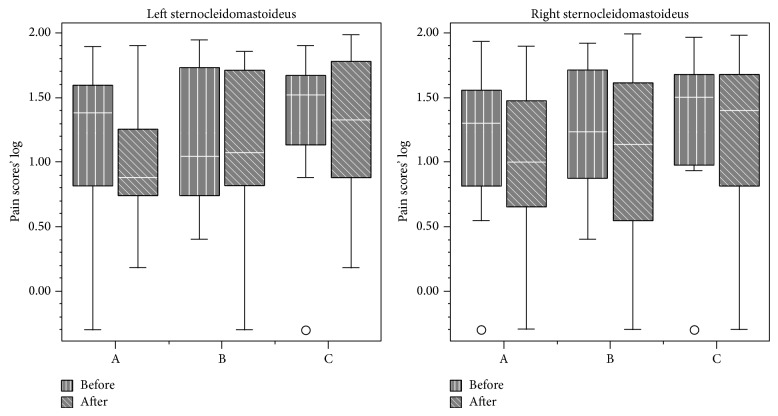
Pain scores (median and interquartile intervals) before and after treatment in the sternocleidomastoideus muscle from groups A (NSAID—sodium diclofenac), B (panacea—sodium diclofenac + carisoprodol + acetaminophen + caffeine), and C (placebo).

**Figure 4:**
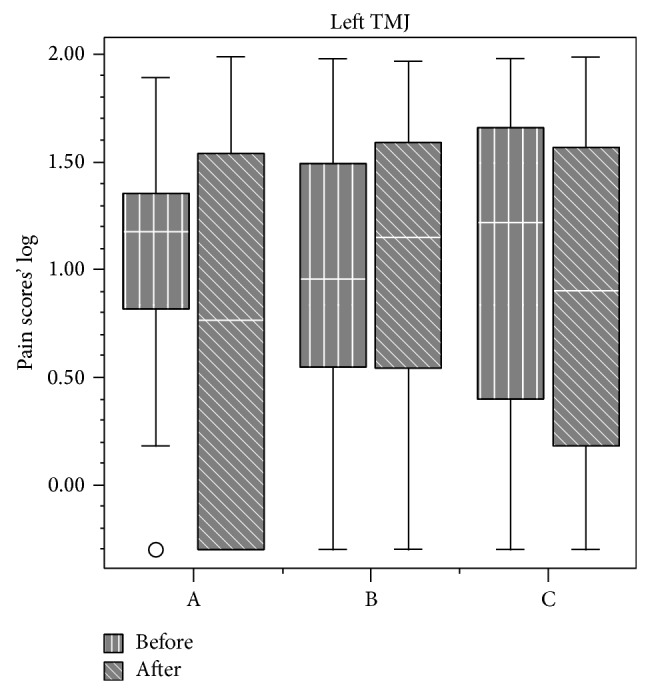
Pain scores (median and interquartile intervals) before and after treatment in the temporomandibular joint from groups A (NSAID—sodium diclofenac), B (panacea—sodium diclofenac + carisoprodol + acetaminophen + caffeine), and C (placebo).

Data from VAS analysis are displayed in Figures 1, 2, 3, and 4. First and final values among evaluated groups did not show significant differences (*P* > 0.05), except for right masseter muscle, in which differences were observed between groups A and C (*P* < 0.05).

When data from 11-point numeric scale was evaluated (daily pain scores), it significant differences were observed between all sites after treatment protocols (*P* < 0.05), except for right TMJ and right/left temporalis muscle (*P* > 0.05).

For A treatment (NSAID—sodium diclofenac), Friedman's test showed significant differences among daily pain scores (*P* = 0.00002). Wilcoxon's test results revealed differences between days 1 × 3 (*P* = 0.019), 1 × 4 (*P* = 0.019), 1 × 6 (*P* = 0.026), 1 × 8 (*P* = 0.006), 1 × 9 (*P* = 0.021), and 1 × 10 (*P* = 0.022).

Regarding B treatment (panacea—sodium diclofenac + carisoprodol + acetaminophen + caffeine), Friedman's test showed significant differences among daily pain scores (*P* = 0.00016). Wilcoxon's test results showed significant differences between days 1 × 3 (*P* = 0.018), 1 × 4 (*P* = 0.027), 1 × 5 (*P* = 0.002), 1 × 7 (*P* = 0.012), 1 × 9 (*P* = 0.004), and 1 × 10 (*P* = 0.011).

About C treatment (placebo), Friedman's test indicated significant differences among daily pain scores (*P* = 0.012). Wilcoxon's test results showed significant differences only in days 1 × 8 (*P* = 0.025).

## 4. Discussion

Analgesic therapies, mainly those based on drugs administration, have been used extensively for the control and treatment of pain, but it is still lacking or scarce in the current literature data on the effects of NSAID associated with occlusal split and other therapeutic agents on the pain relief of patients with chronic muscle pain. In this investigation, we evaluated the efficacy of NSAID, associated or not with therapeutic agents, in the adjuvant treatment of chronic muscle pain. A placebo was used as negative control.

Overall, our findings demonstrated that all the therapies were effective in pain relief. NSAID therapy promoted analgesia on the third day, while placebo promoted analgesia only in the eighth day. No significant differences between medicaments were observed for VAS analysis, except for the right masseter. Daily analysis by 11-point scale showed significant differences between all groups during the 10 days for all sites, except for the right TMJ and right/left temporalis muscle.

The diagnosis method using RDC/TMD and the well-controlled experimental design with inclusion/exclusion criteria associated with the triple-blind randomized crossover clinical methodology were essential to minimize clinical research bias [13–16]. The placebo group, used as control, was fundamental to compare pain relief induced by tested medicaments. Occlusal splints were important for this study to become ethically practicable. Thus, even patients in placebo treatment group received a widely accepted therapy [13, 17, 18].

Our data are in accordance with other similar studies. In a systematic review [19], scientific evidences about splint therapy benefits in TMD symptoms remission after treatment with stabilizing splints were observed. In a study with electromyography [17], immediate decreasing in masseter and temporalis muscle activity after occlusal splints installation was observed, reducing TMJ overload, balancing masticatory muscles, and reducing TMD symptoms. In another study, in which patients were submitted to splint therapy during 1 year, they have reported a relevant decrease in the TMD symptoms, including TMJ sounds [18].

Chronic musculoskeletal pain treatment is complex, and its condition includes inflammatory and noninflammatory characteristics. Some events as repetitive overloading and trauma are clearly associated with peripheral tissue damage and muscle inflammation [7, 9]. Specifically, in masticatory muscles, pain can be a result of occlusal dysfunction and parafunctional habits. Prolonged and repetitive muscular contractions can be aggressive, causing microscopic tissue lesions, sensibility, and pain [20]. In this case, the NSAID could be helpful do reduce or eliminate local inflammation and pain.

The initial hypothesis of this study was that a NSAID (sodium diclofenac) could minimize or inhibit the local releasing of inflammatory substances in many of the masticatory muscles [5] and TMJ pain conditions [21]. In addition, when associated with other substances, such as muscle relaxant (carisoprodol), analgesic (acetaminophen), and central analgesic and adjuvant (caffeine), the effects could be improved.

Provoked pain analysis showed pain reduction on the right and left masseter, sternocleidomastoideus, trapezius, and left TMJ for all treatments. Thus, they were significantly effective in reducing pain sensation, but medicaments A and B were not more effective than placebo after 10-day treatment.

Pain relief could be related to reduction of peripheral sensitization by decreasing of overload, fatigue, and inflammatory components release in masticatory muscles and TMJ [22, 23]. Therefore, allodynia and hyperalgesia could be decreased, as well as painful sensation. The symptom remission could be attributed to splint therapy because, after treatment, results were similar even when using medicaments as adjuvant.

However, occlusal splint associated with medicaments A or B reduced pain in an earlier moment (third day) when compared to control group (eighth day); it has been evident after patients' daily pain scores analysis. Thus, sodium diclofenac, whether associated or not, was shown to be more effective in reducing inflammation process from masticatory muscles and tissue microlesions of TMJ, when compared to placebo [24]. Its action, based on cyclooxygenase inhibition, has prevented development of inflammatory substances from arachidonic acid [23].

Apparently, the use of occlusal splint reduced functional overload on masticatory muscles and TMJ, promoted bilateral balancing, and diminished muscle and joint activity [17] thus, preventing new tissue microlesions and inflammatory mediators release. Concomitantly, the anti-inflammatory acted as inhibiting inflammatory mediators release and reducing peripheral sensitization and, consequently, painful sensation in a short time.

Standardized volunteers' selection based on their TMD signs and symptoms using RDC/TMD was possible, although psychosocial factors could have great variation among them [4]. It means that similar dysfunctions can present different responses, according to patients' mental and social health. Those factors have great influence on pain behavior and should always be considered in chronic pain management. Treatment based on biomedical model, ignoring psychosocial characteristics, may lead to failure and patient's frustration [25, 26]. Nevertheless, an adequate initial approach is essential for treatment success.

Even in chronic pain patient who, according to biopsychosocial pain model, usually has no notable sensorial component when compared to amount of psychosocial factors, the inflammation management using medicaments showed in our investigation significant results in pain relief.

Although participants in this study were chronic TMD patients, with painful complaints strongly influenced by psychological and social status, treatments were based on noxious input decreasing, by drug intake and use of occlusal splint, but analgesia was obtained in a short time and could have a positive effect on patients expectations related to treatment, making them believe in good results, changing their behavioral in regard to their problems, and improving the psychosocial pain components [27].

Management of those drugs in a short period is important to avoid gastric, intestinal, renal, and hepatic problems, as described in the literature [9]. All NSAIDs have gastrointestinal risks of gastritis and possible bleeding and its chronic use increases the risk of renal insufficiency, especially patients with diabetes [22].

Acetaminophen is usually well tolerated at therapeutic doses but may have chronic renal or hepatic adverse effects [27]. Muscle relaxant side effects include high incidence of dizziness, drowsiness, headache, blurred vision, nausea, and vomiting and must be used with caution [28].

Carisoprodol abuse is reported in the literature [29], and its excessive use can cause physical and psychological dependence [28]. Nevertheless, no additional benefit was observed with sodium diclofenac associated with carisoprodol, caffeine, and acetaminophen. Conversely, the literature reports on carisoprodol abuse and dependence and presence of side effects as sleepiness and others related to central nervous system, such as drowsiness, dizziness, palpitation, irritation, and headaches [30]. Clinicians must be alerted to the medicament risks and benefits before prescribing it to patients.

Management of chronic pain is not frequently limited to pharmacotherapy and splint therapy. It should be multidisciplinary, focusing all pain aspects according to biopsychosocial model. The correct and effective intervention of patients suffering of pain could significantly increase their confidence on the proposed therapies. In such case, it seems that starting a treatment with occlusal splint associated to an adjuvant nonsteroidal anti-inflammatory for a short period could be very adequate.

## 5. Conclusion

Within the limitations of this investigation, we conclude that (1) all proposed therapies evaluated reduced TMD pain after 10 days of treatment and (2) NSAID (sodium diclofenac) and panacea (sodium diclofenac + carisoprodol + acetaminophen + caffeine) were more effective than placebo, promoting significant pain relief on the third day, while placebo group promotes only on the eighth day.

## Figures and Tables

**Table 1:** Composition, active ingredients, concentration, manufacturer, and lot number of drugs used in the study.

Medicament	Active ingredient	Concentration	Manufacturer	Lot number
A Register number 1.0370.0150.003-6	Acethaminophen	300 mg	Teuto Laboratory	183567
	Sodium diclofenac	50 mg	(Anápolis, GO, Brazil)	
	Carisoprodol	125 mg		
	Caffeine	30 mg		

B	Sodium diclofenac	50 mg	Teuto Laboratory	40284
Register number 1.0370.0070.003-7			(Anápolis, GO, Brazil)	

C	Cornstarch	110 mg	Faculty of Pharmaceutical	
Placebo (control)			Sciences of Ribeirão Preto of USP	
